# Use of ECMO in ARDS: does the EOLIA trial really help?

**DOI:** 10.1186/s13054-018-2098-6

**Published:** 2018-07-05

**Authors:** Luciano Gattinoni, Francesco Vasques, Michael Quintel

**Affiliations:** Department of Anesthesiology, Emergency and Intensive Care Medicine, University of Göttingen, Robert-Koch-Straße, 40, 37075 Göttingen, Germany

Since 1979, four randomized trials have studied the effectiveness of extracorporeal membrane oxygenation (ECMO) in respiratory failure. Zapol et al. found no difference between patients treated with mechanical ventilation (MV) alone and patients treated with MV plus ECMO [1]. The groups differed with regard to the use of ECMO and FiO_2_, without changing the mechanical ventilation variables. Fifteen years later, Morris et al. compared conventional MV, and low frequency MV combined with mid-flow extracorporeal CO_2_ removal [2]. Both studies suffered from technical difficulties. The technology was fairly primitive then, but has since steadily improved. Peek et al. (CESAR trial) studied patients with severe respiratory failure treated either with MV plus ECMO in a specialized center or with conventional MV in peripheral hospitals [3]. The combined primary outcome parameter, mortality or severe disability after 6 months, was better in the ECMO group. However, of the 90 patients assigned to ECMO only 68 were actually connected, and the study design itself raised concerns about the robustness of the conclusions [3].

The results of EOLIA (ECMO to Rescue Lung Injury in Severe ARDS), a new multicenter randomized controlled trial (RCT), are now available [4]. The primary aim was to investigate whether veno-venous ECMO combined with conventional MV gives a different outcome to MV alone. The entry criteria were severe hypoxemia (PaO_2_ < 60 or 80 mmHg for 3 or 6 h, respectively) or hypercapnia (PaCO_2_ > 60 mmHg or pH < 7.35 for at least 6 h). Crossover from the control arm to ECMO treatment was allowed if prolonged periods of arterial oxygen desaturation to < 80% occurred. The study was terminated for futility after 67 months. There was an 11% reduction in absolute 60-day mortality in favor of ECMO (35 vs 46%), but this difference failed to reach statistical significance (*p* = 0.07). During this period, 249 patients had been enrolled in 64 units, corresponding to an average enrolment rate of 0.058 patients/unit/month (i.e., less than 1 patients/unit/year). Noteworthy is that 35 patients (28%) of the control group required emergency cross-over to ECMO. The patients on ECMO had a significantly higher incidence of bleeding events requiring transfusion (46 vs 28%) and severe thrombocytopenia (27 vs 16%).

We believe that two aspects of EOLIA deserve closer consideration: results and feasibility and utility of ECMO trials.

The results of this study should have proved or disproved two hypotheses:

- *Emergency ECMO improves outcome by “buying time” in extremely hypoxemic patients*.
  Of the 35 patients switched from conventional therapy to rescue ECMO (median SaO_2_ 77%; nine cardiac arrest events), 15 survived. It is unlikely that they would have survived without ECMO, regardless of the statistical relevance of these observations.
- *ECMO improves outcome by reducing the invasiveness of mechanical ventilation.*
  During ECMO, tidal volume was reduced by 43% and respiratory rate by 23%, while PEEP remained essentially unchanged. This represents an estimated 66% reduction in the mechanical power applied to the lungs (from 28 J/min to 10 J/min). This reduction was associated with a higher survival rate (81/124 patients) in the ECMO group (vs 68/125 controls).

But the authors of the study came to the following conclusion: “Among patients with very severe ARDS, 60­day mortality was not significantly lower with ECMO than with a strategy of conventional mechanical ventilation that included ECMO as rescue therapy”. However, taking the cross-over rate into account, and considering that the survival rate of the crossed-over patients without ECMO would have been between 0 and 33%, one finds a significant increase in relative risk reduction with ECMO (from 0.74 to 0.62, *p* < 0.001 and *p* = 0.045, respectively) compared to the relative risk reduction of 0.76 (*p* = 0.09) actually found with the conventional intention-to-treat analysis.

The study has obvious limitations, which primarily reflect the logistic and clinical difficulties in conducting an RCT in this field. These include the extremely low enrolment rate and the unexpectedly high rate of cross-overs from the control group. Nonetheless, we find the results quite impressive. An 11% absolute mortality reduction in patients receiving ECMO is remarkable. Are we certain that a relative risk of 0.76 is really that different from one of 0.74, and that the statistical uncertainty reflected by a *p* value of 0.07 is so different from that of the magic 0.05? Indeed, after reading the paper and supplement with a focus on survival rates and statistical significance, our conclusions might differ from those of the authors. These results will split clinicians into believers and non-believers, and may prompt a request for another high-quality RCT. It is our strong belief that this is simply not possible [5].

## Rationale, feasibility, and utility of RCTs of ECMO vs MV

As a rescue in the emergency setting, an RCT of ECMO versus non-ECMO in severe hypoxemia is ethically unacceptable and comparable to one in situations such as CPR. Instead, comparing ECMO to non-ECMO to decrease ventilator-induced lung injury in stable ARDS requires first a realistic estimate of the mortality attributable to mechanical ventilation. EOLIA was planned assuming that protective mechanical ventilation per se has a 20% mortality, while ECMO itself has a zero risk of mortality. Negative trials do not indicate whether a given intervention is useless, but simply fail to confirm the hypothesis [6]. Indeed, a reduction of 20% in absolute mortality is, in our opinion, an unrealistic goal, whereas the observed recruitment rate of 0.06 patients/unit/month represents real world conditions. Calculations based on the characteristics of the patients in EOLIA show that 624 patients would be required for a study with sufficient power to detect a significant 11% absolute mortality reduction in ECMO from 46% mortality of non-ECMO patients. With the enrolment rate of the CESAR trial (0.03 patients/unit/month) or the EOLIA trial (0.058 patients/unit/month) and 100 participating units, such a study would take 17 or 9 years, respectively.

And finally, will this study change clinical practice or the incidence of ECMO use? Looking back on 50 years of ARDS and 40 years of ECMO history, this seems rather unlikely, since this study yet again provides data for the weal and woe of ECMO just as the previous studies did. Statistics are an operational tool and not a religion; the knowledge, skill, and common sense of physicians are the values in the balance with “0.05”.
